# Supplementary material for: A Comparison of Magnetic Resonance Imaging Methods to Assess Multiple Sclerosis Lesions: Implications for Patient Characterization and Clinical Trial Design
Source: Diagnostics (Basel). 2021 Dec 30;12(1):77. doi: 10.3390/diagnostics12010077 (PMC8775217; doi:10.3390/diagnostics12010077)
Supplement: Supplementary file 1 [file diagnostics-12-00077-s001.zip › diagnostics-1469794-supplementary.pdf]

diagnostics

MDPI

Supplementary Material

Table S1: Subject demographics: TP = Time point: EDSS = Expanded Disability Status Scale. Note, the number of QSM+ lesions changed (from 12 to 14) for case 10, no others changed.

ID	Age	Sex	EDSS TP1	EDSS TP2	EDSS change	# QSM+ lesions	Medications
1	35	F	2.5	3	0.5	61	Tysabri
2	30	F	0	1.5	1.5	19	Tecfidera
3	24	F	1	1.5	0.5	25	Gilenya
4	35	F	2	2	0	7	Amantadine, Baclofen
5	38	F	1	1.5	0.5	35	Tecfidera
7	35	M	1	1	0	12/14	Copaxone
8	51	F	3	3	0	29	Copaxone
9	39	F	1	1	0	0	Rebif
10	37	M	0	1.5	1.5	46	Tecfidera
11	35	F	2.5	2.5	0	24	Gilenya
12	26	F	0	1	1	4	Cell cept
13	31	F	1.5	1.5	0	10	Tecfidera
14	55	F	3	3	0	0	Gabapentin
15	54	F	4	3.5	-0.5	5	Copaxone
16	36	F	2	2	0	33	Tecfidera
17	48	M	2	3.5	1.5	33	Tecfidera
18	37	F	1	2	1	6	Copaxone
19	37	M	1.5	2.5	1	19	-
20	39	M	6	6	0	6	Tysabri
21	33	F	1.5	3	1.5	6	Copaxone

Table S2: Imaging parameters for the MRI protocol

Sequence Name	Low FA SWI	High FA SWI	T2 FLAIR	T2 TSE	MTC	Pre/ post T1 MP RAGE	MWF	DTI
**Orientation**	Axial	Axial	Axial	Axial	Axial	Axial	Axial	Axial
**Acquisition type**	3D	3D	3D	2D	3D	3D	3D	2D
**TR (ms)**	25	25	6000	5100	37	1680	650	8400
**TE (ms)**	7.5, 17.5	8.75, 18.75	397	80	11	3.61	18 ech-oes	107
**TI (ms)**	N/A	N/A	2200	N/A	N/A	900	N/A	N/A
**FA (degree)**	6	24	N/A	120	15	9	90	N/A

**Base Resolution**	384	384	256	384	384	512	160	96
**FOV phase (%)**	75%	75%	100%	75%	100%	100	75%	100%
**Matrix size**	144 x 384	144 x 384	256 x 256	144 x 384	144 x 384	256 x 512	192 x 256	96 x 96
**FOVphase x FOVread**	192 x 256	192 x 256	192 x 256	192 x 256	192 x 256	256 x 256	120x 160	256 x 256
**Slice Gap %**	-	-	N/A	0%	20%	0%	50%	0
**TH (mm)**	2.0	2.0	1.34	2	2.7	1	5	2.7
**No of Slices**	80	80	80	80	80	160	24	56
**Voxel size (mm^3)**	1.33x0.67x2.0	1.33x0.67x2.0	1.33x0.67 x1.34	1.33x0.67x2.7	1.33x0.67x2.7	1.0×0.5 ×1.0	1.6 x 1.6 x 5	2.7 x 2.7 x 2.7
**Interpolation**	OFF	OFF	ON	OFF	OFF	OFF	OFF	OFF
**#DTI dir**	N/A	N/A	N/A	N/A	N/A	N/A	N/A	30
**measurements**	1	1	1	1	1	1	1	N/A
**Concatenations**	1	1	1	3	1	1	1	1
**# Acq/averages**	1	1	1	1	1	1	1	1
**Fat Suppr**	None	None	None	None	None	None	Fat Sat	Fat Sat
**Water Suppr**	None	None	None	None	None	None	N/A	N/A
**Phase resolution**	50%	50%	50%	50%	50%	50%	100%	100
**Slice Resolution**	100%	100%	100%	N/A	100%	75%	100%	N/A
**Slice Oversampling**	10%	10%	0	N/A	0%	0%	0%	N/A
**Phase Oversampling**	0.00%	0.00%	100%	0%	0%	0%	0%	0
**Phase Enc. Dir**	R>>L	R>>L	R>>L	R>>L	R>>L	A>>P	R>>L	A>>P
**Accel. factor PE2**	1	1	1	N/A	N/A	1	N/A	N/A
**Accel. factor PE**	2	2	2	2	2	2	N/A	2
**Partial Fourier (phase)**	OFF	OFF	7/8	OFF	OFF	OFF	OFF	OFF
**Partial Fourier (Slice)**	OFF	OFF	7/8	OFF	OFF	OFF	3/4	N/A
**Ref. lines PE**	24	24	24	35	24	24	N/A	40
**BW (Hz/Px)**	270	270	781	221	70	180	976	1860
**Flow Comp**	yes	yes	no	no	no	slice	N/A	N/A
**Echo train per slice**	N/A	N/A	1	5	N/A	N/A	N/A	N/A
**b - Value s/mm²**	N/A	N/A	N/A	N/A	N/A	N/A	N/A	0, 1000, 2000
**Slice Turbo/Turbo/ EPI Factor**	N/A	N/A	2, 141, N/A	N/A, 18, N/A	N/A	N/A	N/A, N/A,3	N/A, N/A, 80
**Scanner Acquisition Time (sec)**	3:04	3:04	5:14	3:09	5:13	3:55	7:48	9:02

Table S3: Quantitative susceptibility map (QSM) positive and negative lesions and normal appearing white matter (NAWM) results. TP: represents time point.

Lesions							
**Sequences**	**QSM positive (18 subjects)**						
	**Mean lesion volume (mm³)**		**Mean Signal**		**Mean SD**		**Z-score**
	**TP1**	**TP2**	**TP1**	**TP2**	**TP1**	**TP2**	
**T2 FLAIR**	553	474	215	216	18	18	-0.54
**T2**	422	431	853	861	115	116	-0.70
**MTR**	302	310	0.32	0.32	0.03	0.03	-0.10
**QSM**	213	193	18	18	14	13	0.10
**FA**	239	238	0.35	0.35	0.09	0.09	-0.04
**ADC**	273	266	0.93	0.96	0.14	0.15	-0.40
**RD**	272	267	0.58	0.59	0.12	0.12	-0.18
**MWF**	247	231	8.26	8.66	3.67	3.63	-0.24
**STAGE T1MAP**	380	400	1431	1427	307	299	0.15
**STAGE PSDMAP**	379	401	2482	2496	213	228	-0.63
**Sequences**	**QSM negative (11 subjects)**						
	**Mean lesion volume (mm³)**		**Mean signal**		**Mean SD**		**Z-score**
	TP1	TP2	TP1	TP2	TP1	TP2	
**T2 FLAIR**	478	433	206	212	25	26	-0.40
**T2**	452	417	788	801	91	100	-1.39
**MTR**	274	273	0.35	0.34	23	24	0.40
**QSM**	365	347	-9	-9	12	12	0.10
**FA**	252	238	0.34	0.32	0.09	0.09	0.41
**ADC**	259	241	0.90	0.94	0.15	0.14	-0.46
**RD**	260	241	0.55	0.57	0.11	0.11	-0.38
**MWF**	374	362	10.65	9.96	3.59	3.21	0.54
**STAGE T1 Map**	394	369	1336	1367	229	244	-1.29
**STAGE PSD Map**	394	369	2490	2501	188	195	-0.56
**NAWM**							
**Sequences**	**MS (contralateral to lesions)**						
	**Mean Vol. of ROI (mm³)**		**Mean signal**		**Mean SD**		**Z-score**
	**TP1**	**TP2**	**TP1**	**TP2**	**TP1**	**TP2**	
**T2 FLAIR**	268	305	159	154	9	9	1.14
**T2**	289	313	548	545	39	37	0.56

**MTR**	265	253	0.41	0.41	0.01	0.01	-0.01
**QSM**	169	157	-7	-6	9	9	0.08
**FA**	186	177	0.62	0.63	0.10	0.10	-0.25
**ADC**	204	190	0.68	0.70	0.09	0.09	-0.37
**RD**	204	190	0.34	0.33	0.07	0.07	0.26
**MWF**	221	205	14.77	15.77	3.61	3.74	-0.55
**STAGE T1 Map**	262	293	940	940	100	90	0.02
**STAGE PSD Map**	262	293	2077	2082	98	99	-0.45
**Sequences**	**HC (drawn across 4 different brain regions)**						
	**Mean Vol. of ROI (mm^3)**		**Mean signal**		**Mean SD**		**Z-score**
	**TP1**	**TP2**	**TP1**	**TP2**	**TP1**	**TP2**	
**T2 FLAIR**	679	723	152	151	15	16	0.93
**T2**	679	723	502	505	50	47	-1.04
**MTR**	386	420	0.40	0.41	0.02	0.02	0.00
**QSM**	368	406	-8	-8	8	9	0.19
**FA**	145	193	0.57	0.57	0.15	0.14	-0.27
**ADC**	145	193	0.69	0.68	0.10	0.12	0.59
**RD**	145	193	0.34	0.33	0.09	0.08	0.60
**MWF**	325	294	15.94	17.64	4.33	4.13	-3.57
**STAGE T1 Map**	622	654	998	1003	126	117	-0.45
**STAGE PSD Map**	622	654	2199	2115	109	84	0.83
